# Looping Flexible Fluoropolymer Microcapillary Film Extends Analysis Times for Vertical Microfluidic Blood Testing

**DOI:** 10.3390/s24185870

**Published:** 2024-09-10

**Authors:** Rüya Meltem Sarıyer, Kirandeep K. Gill, Sarah H. Needs, Nuno M. Reis, Chris I. Jones, Alexander Daniel Edwards

**Affiliations:** 1 Reading School of Pharmacy, University of Reading, Whiteknights, Reading RG6 6UB, UK; r.sariyer@pgr.reading.ac.uk (R.M.S.); s.h.needs@reading.ac.uk (S.H.N.); 2 Department of Surgery, Center for Engineering in Medicine and Surgery, Massachusetts General Hospital, Harvard Medical School, Boston, MA 02129, USA; kgill7@mgh.harvard.edu; 3 Department of Chemical Engineering and Centre for Bioengineering and Biomedical Technologies (CBio), University of Bath, Bath BA2 7AY, UK; n.m.reis@bath.ac.uk; 4 Reading School of Biological Sciences, University of Reading, Whiteknights, Reading RG6 6AH, UK; c.i.jones@reading.ac.uk; 5 School of Electronics and Computer Science, University of Southampton, Highfield, Southampton SO17 1BJ, UK; 6 Institute for Life Sciences, University of Southampton, Highfield, Southampton SO17 1BJ, UK

**Keywords:** microfluidics, loop, capillary rise, flow dynamics, Raspberry Pi, coagulation, blood analysis

## Abstract

The microfluidic measurement of capillary flow can be used to evaluate the response of biological samples to stimulation, where distance and velocity are altered. Melt-extruded multi-bored microfluidic capillaries allow for high-throughput testing with low device cost, but simple devices may limit control over sample flow when compared to the more complex “lab-on-a-chip” devices produced using advanced microfluidic fabrication methods. Previously, we measured the dynamics of global haemostasis stimulated by thrombin by dipping straight vertical microcapillaries into blood, but only the most rapid response could be monitored, as flow slowed significantly within 30 s. Here, we show an innovative method to extend both the stimulation process and flow measurement time without increasing the cost of the device by adding simple loops to the flexible extruded device. The loops enable longer time-scale measurements by increasing resistance to flow, thereby reducing the dependence on high stimulus concentrations for rapid reactions. The instantaneous velocity and equilibrium heights of straight and looped vertical microcapillary films were assessed with water, plasma and whole blood, showing that the loops create additional frictional resistances, reduce flow velocity and prolong residence times for increased time scales of the stimulation process. A modified pressure balance model was used to capture flow dynamics with the added loop. Looped devices loaded with thrombin and collagen showed an improved detection of blood stimulation responses even with lower stimulus concentrations, compared to straight vertical capillaries. Thrombin-activated blood samples in straight capillaries provided a maximum measurement zone of only 4 mm, while the looped design significantly increased this to 11 mm for much longer time scale measurements. Our results suggest that extending stimulation times can be achieved without complex microfluidic fabrication methods, potentially improving concentration–response blood stimulation assays, and may enhance the accuracy and reliability. We conclude adding a loop to low-cost extruded microfluidic devices may bring microfluidic devices closer to delivering on their promise of widespread, decentralized low-cost evaluation of blood response to stimulation in both research and clinical settings.

## 1. Introduction

Intense research on the production of microfluidic technology from the 1990s [1] has led to explorations across a wide range of applications, particularly in the medical, biological, and chemical measurement fields [2,3]. Microfluidic devices have the potential to reduce testing costs, smaller test volumes can reduce the consumption of reagents, and faster assays can reduce analysis times, alongside increasing portability [1,4]. Miniaturization makes it possible to create portable devices for on-site testing that can be used in various applications, including micro-total analysis systems, lab-on-a-chip, actuators, and miniaturized point-of-care devices [3]. The need for precise manufacturing processes for more complex microfluidic systems with sub-millimetre length-scale channels comes at a cost, leading to the pursuit of alternative fabrication processes for micro-scale devices, including melt extrusion that produce capillaries at very low costs, as well as lamination and paper-based formats. Despite the numerous potential advantages of microfluidic devices, the satisfactory release of reagents required for various purposes, such as diluting samples, homogenizing reagents and facilitating chemical or biological reactions, may not be as simple to achieve as traditional manually operated biological or chemical assays [4,5]. Obtaining the optimal release and mixing of reagents is critical [6] but can be difficult to achieve in sub-millimetre dimension channels within microfluidic devices due to laminar flows resulting from a low Reynolds number, with diffusion dominating rather than turbulence [4,7]. Bends and curves are typically designed in microfluidic devices to create secondary flows and thereby improve the mixing and transport of fluids or particles within microfluidic channels. Although curves and bends are useful to enhance mixing and promote mass transport within integrated microfluidic devices [8], straight extruded capillaries lack curves, and thus a trade-off is presented between the low-cost and simplicity of extruded devices vs high performance, with complex fluidics possible with more expensive manufacturing methods. To resolve this, if manufactured from flexible materials (e.g., polymers rather than glass) then extruded capillary microfluidic devices can be bent to alter flow and thereby change the fluid dynamics. This was recently exploited to alter biocatalytic reactor properties using simple 3D printed frames to introduce controlled curvature within extruded microcapillaries [6].

Numerous microfluidic devices have been developed to measure aspects of blood function. Microfluidic devices allowed for the measurement of platelet aggregation [9,10,11] and blood coagulation [12] based on migration distance, as well as ABO blood group typing [13]. Additionally, the existing literature review showed that blood plasma separation devices have been constructed using different channel geometries, including T-channels, Y-channels, bend channels, curved channels, spiral channels, and serpentine channels [14]. Many researchers [15,16,17,18,19,20,21,22] have favoured bend, curved, spiral, and serpentine channel geometries for both diluted and undiluted blood separation [14]. However, such channel geometries are commonly utilized in separation techniques using filtration or centrifugation, as well as exploiting hemodynamic effects in some cases [14]. Microfluidic devices allow for the precise manipulation of fluid flow, enabling the creation of well-controlled and reproducible experimental conditions [23]. Platelets are anucleate cells in the blood that primarily function to stop bleeding by forming aggregates for haemostatic reactions; moreover, platelet aggregation is implicated in various diseases or medical conditions, such as inflammation, atherosclerosis, and cancer metastasis [24]. The time it takes for platelet agonists to activate the platelets may depend on several factors, such as the type and concentration of the agonist and the presence of other mediators [24]. Thus, one of the key properties of microfluidic devices for platelet function assays is the ability to control the incubation times of platelet agonists.

In our previous study, we studied blood rise in straight vertical microcapillaries [25]. The flow analysis of individual capillaries with time and distance resolution was performed by time-lapse digital imaging. Time-resolved capillary rise monitoring provided quantitative information on fluid properties, such as blood after stimulation. Global haemostasis activation was triggered by stimulating blood with thrombin, and dynamic measurements showed that vertical fluid velocity slowed down in accordance with the increase in viscosity. The short incubation times of platelets within these microcapillary film ‘dip-stick’ tests can limit the sensitivity and precision of such tests. In these straight vertical devices, the blood rises rapidly within the first 20 s, but then slows [25], limiting the time period where the response of blood to agonists can be monitored through flow properties. This may explain why the clearest responses observed with straight vertical capillaries were seen at highest thrombin concentration slows [25]. Several important blood stimulation agents trigger slower responses, which would be hard to measure within a 20–30 s stimulation [26]. As vertical flow slows, it becomes harder to measure fluid properties such as viscosity. By designing microfluidic devices that allow for the extended incubation times of platelets, it should be possible to overcome these limitations, ideally maintaining the simple melt-extrusion manufacturing process.

Here, for the first time, we present a novel method where the simple reshaping of extruded fluoropolymer microcapillary film (MCF)—exploiting the flexibility of the film—may enhance the determination of even small differences in blood flow properties after stimulation by the addition of agonists of coagulation and platelet activation during capillary flow. This innovation enables longer time-scale measurements compared to previous studies, which were limited to fast reaction rates and high chemical concentrations. While traditionally microfluidic devices incorporate constrictions or complex structures to provide higher fluid resistance and flow friction, we have chosen to add loops while maintaining the simplicity of the device manufacturing method. This simple yet effective approach increases the reaction time by increasing the resistance to flow and allows for longer time-scale measurements even with low stimulus concentrations. This method relies on blood flow through a microfluidic device driven by capillary forces, while the novel addition of a loop also introduces curvature within a simple “stimulation zone”, near the inlet, where flow is not measured but blood can respond to mixing with agonists. The measurement of migration distance and the velocity of blood in capillaries after leaving the loops and entering a straight vertical “measurement zone” can then be used to assess haemostatic changes. Simply separating measurement from stimulation within the added loop prolongs assay times. Building on a prior demonstration of the benefits of melt-extruded microcapillary film [27,28] and the recent use of 3D-printed templates to drive mixing within biocatalytic reactors formed from in these extruded devices [6], this study is the first to present “dip-stick” [13] blood coagulation and platelet stimulation tests performed in extruded microcapillary film using a loop design. 

## 2. Materials and Methods

### 2.1. Materials and Test Strip Production

The ultrapure water maker (Elga Veolia pure lab Chorus) was obtained from Elga LabWater (High Wycombe, UK); thrombin from bovine plasma (product T4648), Tween 20 (product P1379), and polyvinyl alcohol (MW 146,000–186,000, >99% hydrolysed), were purchased from Sigma Aldrich (Dorset, UK). The platelet-activating preparation of collagen (product P/N 385, comprising native collagen fibrils (type I) from equine tendons) was purchased from Chrono-Log Corporation (Havertown, PA, USA). The microcapillary film manufactured from fluorinated ethylene propylene was obtained from Lamina Dielectric Ltd. (Billingshurst, UK). HEPES-buffered saline was prepared using components from Fisher BioReagents (Leicestershire, UK).

Test strips were prepared as described previously [13,25]. A microcapillary film with ten capillaries (each with an internal diameter of 200 µm) was internally coated with polyvinyl alcohol and then cut into test strips with lengths of 100, 150 and 160 mm, which were then assembled on a 3D printed frame that introduced one or two loops, with a loop diameter of approximately 14 and 9 mm, respectively, with the length of the looped region being 43 and 58 mm, respectively. These test strips were used to perform experiments to measure the capillary rise of blood components. At later timepoints (10 min), the equilibrium height of the fluids was also measured. To test the response of blood to stimuli, the indicated concentrations of thrombin or collagen were loaded as previously described [13,25], by filling reagents into polyvinyl-alcohol coated test strips which were freeze-dried, leaving dried but soluble stimuli deposited within the test strips. The 100 mm-long stimulus-loaded strips without, or with one or two loops, were then used to activate and measure whole blood and compare how the loops affected blood measurement.

### 2.2. Preparation of Blood Samples

Healthy donors’ whole blood (WB) samples were collected using BD Vacutainer^®^ citrate blood collection tubes (Wokingham, UK) with a 3.2% buffered sodium citrate solution that prevents coagulation. Blood (200 µL) was added to a multiwell strip, into which the microcapillary test strips were dipped. The preparation of platelet-rich plasma (PRP), platelet-poor plasma (PPP) and red blood cells (RBCs) was previously described in detail [25].

### 2.3. Flow Dynamics of Looped Microcapillaries

Samples (ultrapure water, HBS, PPP, PRP, RBCs and WB) were placed under the hydrophilic MCF test strips in a clear flat-bottomed 96-well microplate. Sets of 100 mm straight and 150 and 160 mm looped MCF strips were produced such that after the addition of a loop, they were all the same vertical height, allowing endpoint equilibrium rise to be determined. To obtain precise fluid flow velocity measurements, these devices were fitted in the RMS imaging system, described previously [25], which automates the dipping and time-resolved imaging of vertical fluid flow, using the Raspberry Pi singleboard computer and digital camera platform, widely used to build open-source laboratory hardware [29]. The experimental setup was illustrated in a schematic provided as Supplementary Data (Figure S1). During the experiments, the movement of the fluid in the capillary was observed and recorded with the RMS imaging system at the determined measurement time intervals, by time-lapse image capture. After transferring the images to an external computer, the height of the sample in each image was marked at specific points on the image using ImageJ software (version 1.54; NIH, Bethesda, MD, USA [30]). These heights and timepoints were used to determine the distance the fluid travelled. The obtained height data were converted into experimental velocities by calculating the instantaneous superficial fluid velocity, dH/dt, to determine the fluid’s movement speed, through a comparison of pairs of sequential images. This equation allows for the calculation of the velocity of fluid capillary rise by dividing the change in height (dH) by the elapsed time (dt), thereby quantifying the speed of fluid movement over a specified distance. 

To demonstrate that the loop design does not affect the capillary rise (i.e., adding loops does not alter surface tension and contact angle), the equilibrium height of water, PRP and WB was determined after 10 min for both straight and looped strips. An analysis of capillary rise was carried out by using ImageJ. Subsequently, the instantaneous mean superficial fluid velocity (dH/dt) was estimated from the equilibrium height values of pure water, PRP and WB to determine the effect of adding loops during capillary flow on flow dynamics. These values were used to evaluate the changes in pressure drop and flow velocity caused by the addition of loops.

### 2.4. Analytical Modelling of Fluid Rise in the Straight and Looped Microcapillaries

We have previously modelled [25] the capillary rise of sample liquids in vertical hydrophilic microfluidic strips using a simple pressure balance model that captures both the dynamic flow and the equilibrium state of liquid samples drawn into microcapillaries by capillary action. With straight vertical test strips, the length is equal to the height such that $L\left(t\right)=H\left(t\right)$, and thus the instantaneous fluid rise velocity can be determined as Equation (1):

(1) $$\frac{dH}{dt}=\frac{1}{H(t)}\left[\frac{\left(\frac{1}{a}+\frac{1}{b}\right)\gamma cos\theta -\rho gH(t)}{32\mu }\right]d_{h}^{2}$$

where *a* and *b*, respectively, are the major axis and minor axis of the capillary’s cross-section, γ is surface tension of the liquid, and *θ* is the contact angle between the liquid and the capillary surface. *ρ* is density of the liquid, g is gravitational acceleration and H is liquid height, measured between the liquid level in the sample reservoir and the meniscus. μ is dynamic fluid viscosity, $d_{h}$ is the hydraulic diameter of an ellipse, and L is the total fluid length within the microcapillary.

When loops are added to the vertical strips, the model needs to be adjusted so that the flow resistance term takes into account the additional distance travelled around the loop. When a loop is added to vertical microcapillary strips, the value of L(t) is increased; thus, $L\left(t\right)=H\left(t\right)+L_{loop}$. The revised pressure balance equation states that the total length L(t) is the sum of the height $H\left(t\right)$ and the loop length, $L_{loop}.$ Thus, in the revised configuration, the formula for the looped strips is now represented by Equation (2).

(2) $$\frac{dH}{dt}=\frac{1}{L_{loop}+H(t)}\left[\frac{\left(\frac{1}{a}+\frac{1}{b}\right)\gamma cos\theta -\rho gH(t)}{32\mu }\right]d_{h}^{2}$$

Predicted instantaneous velocities were calculated for test strips with zero, one or two loops using Equation (2) in Excel (Office 365 Excel version 2402), and predicted velocity plots compared to observed flow velocities estimated from sequential pairs of images, using known and predicted fluid properties as described previously [25]. We used a pressure balance model to model the fluid flow and derived an equation of velocity against height by balancing the force of the upward Laplace pressure against downward fluid friction and gravitational forces (Equation (1)). Equation (1) was used to model all the straight capillaries and was adjusted for the length added by the loops to model all the looped microfluidic designs, as shown in Equation (2). These equations were adjusted for fluid properties such as viscosity, density and contact angle to predict velocity changes in different fluids. For pure water, PRP, and WB, known density and viscosity values were used, while surface tension and contact angle were estimated based on equilibrium heights in straight strips. These estimates were performed using Excel’s Solver tool. It was assumed that adding loops would not significantly change the surface properties, and thus these values were applied to looped strips. Additionally, the major axis and minor axis of the capillary’s cross-section, previously derived from microscopic examination of the material [25], were also used in these analyses.

### 2.5. Effect of Loop Addition on Capillary Rise of Blood with Stimulation

To demonstrate the potential for extended incubation times within looped microcapillaries to improve the measurement of stimulated blood flow properties, whole blood samples were tested by dipping thrombin- and collagen-loaded test strips. Vertical flow was recorded by time-lapse imaging using the RMS imaging system. The 100 mm-long test strips were loaded by freeze-drying with thrombin at concentrations of 0, 5, 15, 50, 150 and 300 U mL^−1^ thrombin in water, or loaded with collagen at concentrations of 0, 50, 158 and 500 mg mL^−1^ in water. Reagent loading methods were described previously [25]. To maintain consistent stimulus loading, strips of uniform length were prepared; thus, after creating the loop, the total height was lower. Capillary rise dynamics was monitored for straight and looped strips for up to 2 min. The height *H*(*t*) and length *L*(*t*) of the samples rising within the microcapillaries were analyzed using ImageJ, and pairs of sequential images were compared to estimate instantaneous flow velocity at each height. 

### 2.6. Statistical Analyses 

Statistical analyses were performed using GraphPad Prism version 10.3.0. The normality of the data was assessed using the Shapiro–Wilk test. A value of *p* < 0.05 was used for significance test. One-way ANOVA was performed to compare the effects of adding loops on the height measurements of pure water, PRP, and WB in straight, single loop, and double loop strips. Post hoc analysis using Tukey’s HSD test was conducted to identify significant differences between the groups. 

## 3. Results and Discussion

### 3.1. Loops Introduce Additional Stimulation Zone and Extend Flow Measurement Range 

**Figure 1:**
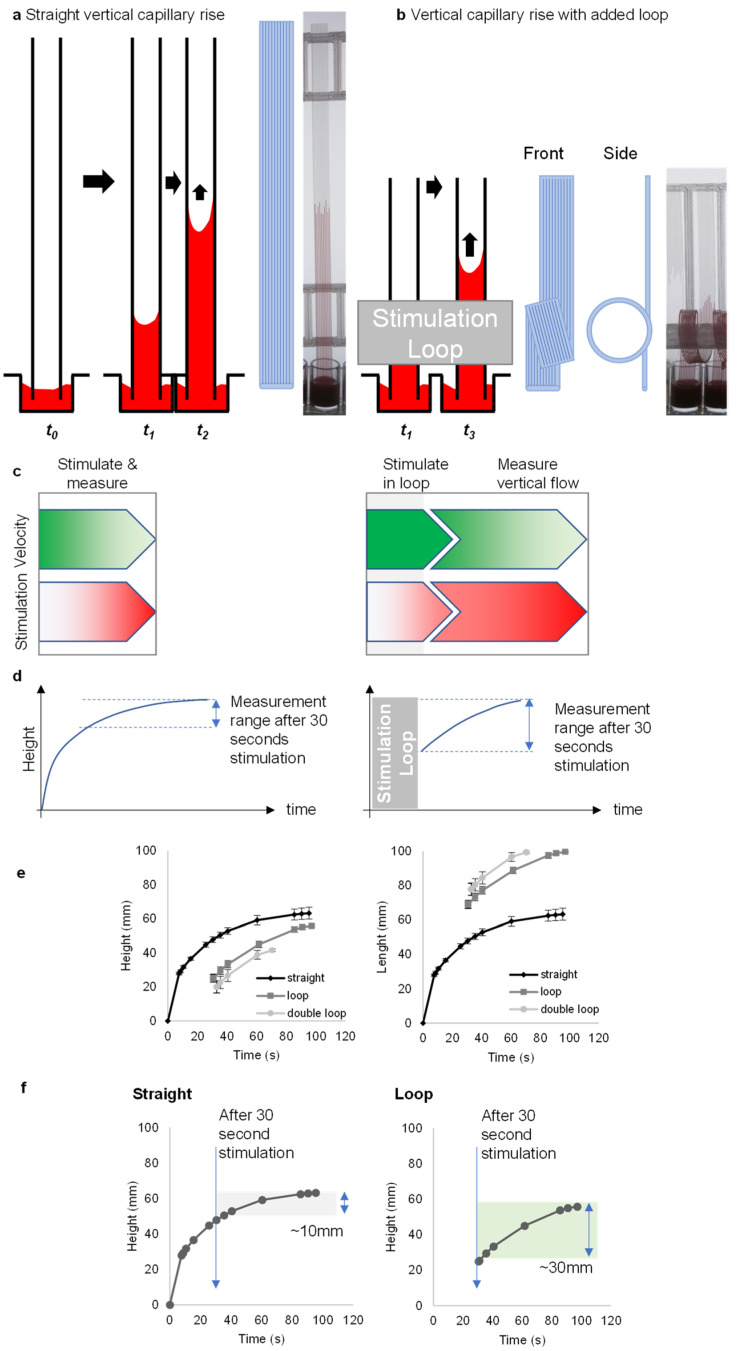
The loop design provides a way of increasing the measurement range by controlling the stimulation time. (**a**) Concept of capillary rise in “dip-and-test” microfluidic straight vertical dipsticks to measure blood function. (**b**) Vertical capillary rise with added loop concept with front and side view. (**c**) The dip-stick tests work by allowing blood samples to flow up vertically into ~200-micron straight channels where the blood mixes with stimuli. When a simple loop is added to the vertical dipstick, blood flows vertically upwards, but then around a loop, and while in that loop it begins to be stimulated. (**d**) The vertical flow in straight channels takes only a few seconds before slowing down. With the addition of a loop, this rapid flow continues for >30 s or so. (**e**) A representation of capillary rise in height and length over time for straight, loop- and double-loop-added dipsticks. Data indicates the mean of 10 replicate capillaries where error bars indicate ± SD from a single donor; similar results were observed in 2 replicate experiments with different donors. (**f**) In loop dipsticks, the blood rises over a greater distance due to the added length of the loop. Straight dipsticks increase by about 10 mm after 30 s, while looped dipsticks achieve around 30 mm within the same time.

Simple ‘dip-and-test’ test strips can make rapid, portable measurements of blood stimulation in straight vertical capillaries (Figure 1a), illustrating the potential for low-cost melt-extruded microfluidic devices to measure blood activation [25]. Here, we show that these microfluidic dipsticks can be significantly improved by adding a simple “loop” to increase the distance travelled and thereby increase the stimulation time. To increase the stimulation time and be able to detect lower concentrations of stimuli in a longer measurement range, we looped the inlet end of stimulus-loaded test strips (Figure 1b). In particular, we found that strips longer than 100 mm caused problems with the freeze-drying process for loading stimuli into test strips. Therefore, we used strips with a maximum length of 100 mm in the stimulation experiments. We use longer strips in the water, plasma and blood experiments without stimuli; these longer strips were used for endpoint measurements after 10 min and were not freeze-dried. We designed a device holder to create loops of standard diameter that wrapped the loops around the ladder. During the initial optimization tests, we found that when more than two loops were added, bends that were too tight caused problems with even flow. Furthermore, when multiple loops made the stimulation length too long, the flow could not be measured because not enough vertical distance was visible after the loops. Based on these initial optimization steps, we chose to contrast straight vertical strips with either single- or double-looped test strips.

Looped test strips sustained flow, and thus the measurement range, after 30 s stimulation, in contrast to straight test strips (Figure 1c,d). Straight strips demonstrate changes only in height, whereas looped strips show changes in both height and length (Figure 1e). The measuring range with straight strips was narrow with only a 10 mm distance of flow after 30 s; this interval was increased to 30 mm after 30 s with the addition of a loop (Figure 1f). Importantly, the loops also permitted the addition of curvature without requiring a different fabrication method, retaining the simplicity and low device cost of melt-extruded test strips.

### 3.2. Capturing Flow Dynamics for Water, Plasma and Blood during Capillary Flow in Vertical and Looped Microcapillaries

To observe how the flow dynamics for different fluids such as pure water, PRP and WB are affected by the addition of loops to the vertical strips, the equation (Equation (1)) obtained in our previous study [25] to calculate the instantaneous fluid rise velocity in capillary flow was rearranged (Equation (2)). We aimed to reduce the number of unknown parameters by fixing the density and viscosity using well-characterized density and viscosity values from the literature for water, plasma (using platelet-rich plasma; PRP) and blood (whole blood; WB). This included known densities (*ρ* = 0.998 g mL^−1^ for water [31], 1.025 for PRP [32] and 1.055 for WB [32]) and expected viscosities (μ = 1.04 cP for water [31], 1.7 for PRP [33] and 6 for WB [34]). We also estimated the surface tension and contact angle values from the equilibrium rise for water, plasma and blood in straight strips [25], and assume that the addition of loops will not change these surface properties.

**Figure 2:**
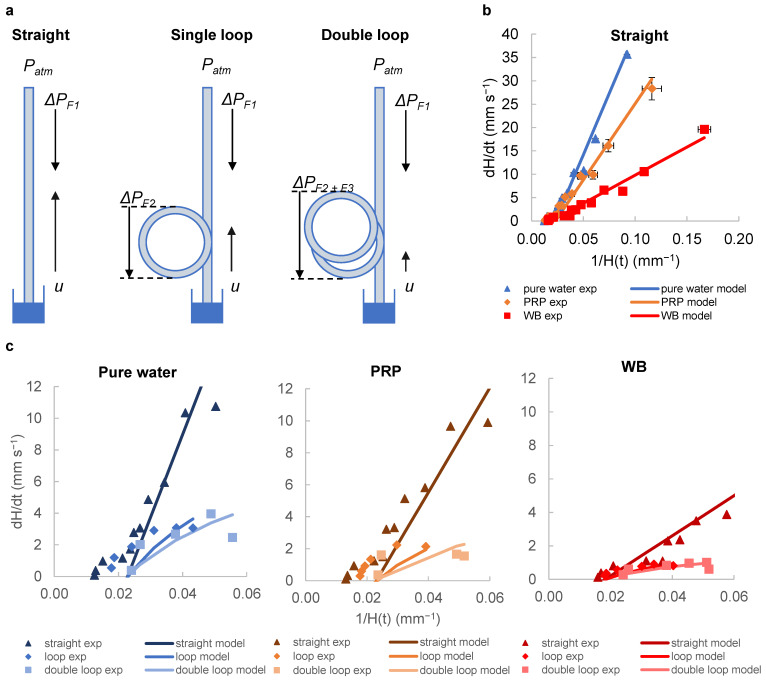
Adding loops to the straight vertical strips creates additional frictional resistances (∆PF). (**a**) ${∆P}_{F1}$ represents the pressure difference driving the flow, while ${∆P}_{F2}$ and ${∆P}_{F2}+{∆P}_{F3}$ represent the additional pressure differences due to the loops. (**b**) The graph shows how the instantaneous superficial fluid velocity dH/dt changes with the reciprocal H(t) for each fluid. The experimental data points are plotted along with the model predictions calculated from the transient pressure balance. The dynamic flows observed for pure water, PRP and WB in straight strips fit our model. Data were collected from 10 replicate capillaries, with error bars representing ± SD from a single donor; consistent results were observed across 2 replicate experiments with different donors. (**c**) Graphs show experimental data points and corresponding model predictions for straight, single-loop and double-loop configurations. Adding loops reduces the flow rate, which leads to longer residence times. The differences in flow rate become more pronounced with different fluid viscosities. Mean of 10 replicate capillaries are shown in the chart, with similar results across 2 independent experiments (N = 2).

Adding loops creates additional frictional resistances (${∆P}_{F}$) (Figure 2a). According to the Hagen–Poiseuille equation, the flow velocity will decrease to maintain the total pressure drop. Therefore, adding loops to the microcapillary strips to extend the distance is expected to lead to higher total pressure drop, and thus lower velocities sustained to later timepoints. We observed good agreement between model predictions and the experimental data points in straight vertical strips for pure water, PRP and WB (Figure 2b). We previously [25] validated the pressure balance model that predicts a linear relationship between dH/dt velocity and reciprocal of liquid head 1/H(t) at early timepoints of capillary rise for straight vertical test strips. Blood and plasma had higher viscosities than water, and these liquids had lower dH/dt values at equivalent heights. 

The imaging setup does not allow for the measurement of flow within the loop, and thus it was only possible to derive height and thus velocity values at later timepoints. We therefore presented the lower velocity ranges corresponding to later times, with 1/H(t) values in the range 0.06–0.0125 mm^−1^ which correspond to heights of 17–80 mm. In this range, there was an increasing discrepancy between prediction and experiment, with consistently lower velocity than that predicted by the simple pressure balance model alone. The pressure balance model used here accounts for the additional length given by the loops, which gives a relatively good estimate with experimental data. To increase the fit, a more complex model is needed that considers other factors, including dynamic changes in the contact angle and possible centrifugal effects. Nevertheless, our model is simple and can be used to quickly and easily design microfluidic capillary devices with desired performance. Key parameters can be planned, such as the optimal height, capillary diameter, and loop length, based on fluid properties and sample behaviour. 

The data show that loop addition affects dH/dt for all fluids tested. Our model confirms the lower flow rates expected with loop addition due to added resistance, and, as planned, resulted in longer sample residence time (Figure 2c). We experimentally observed and modelled the capillary rise of water, PRP and WB in straight and looped capillaries. Adding a single loop resulted in lower velocities for all fluids, and the addition of the second loop decreased the velocities even further. This effect of loops on decreasing velocity was more pronounced for low viscosity fluids such as water (µ = 1.04) compared to high viscosity fluids such as blood, which generally have lower flow rates to begin with (µ = 6). Our pressure balance model was able to predict this flow behaviour relatively well; however, we observed discrepancies between the model and experimental data at higher capillary heights (i.e., lower range of 1/H(t) values). One possible explanation for this divergence could be that the model does not account for fluid flow changes that may occur as the fluid nears the equilibrium height. Especially with high viscosity liquids (e.g., WB), the higher viscous resistance reduces the impact of loops, as the overall velocities are lower. Capillary flow velocity is expected to be directly proportional to surface properties (surface tension and contact angle) and inversely proportional to viscosity; therefore, changes in the properties of liquid samples produce changes in the flow rate in similar ways [35]. Both the single-loop and double-looped capillaries produced similar fluid flow profiles in all fluids tested compared to the straight capillary; the addition of even a single loop has a significant effect on reducing flow, and if even longer stimulation times are required, a double loop becomes beneficial for blood staying in the “stimulation zone” for a longer duration of stimulation. 

Statistical analyses showed that there were significant differences in capillary heights for pure water, PRP and WB samples between the straight, single-loop and double-loop strips measured at 30 s. Normality tests were performed using the Shapiro–Wilk test. The test showed that the data fit a normal distribution (*p* > 0.05). One-way ANOVA analysis then showed that for pure water, there were significant differences between groups (F(2,27) = 368.1, *p* = 0.0001), indicating that the addition of simple loops influenced fluid height measurements. The analyses also showed that there were significant differences between the groups in PRP (F(2,27) = 214.4, *p* = 0.0001) and WB (F(2,27) = 327.5, *p* = 0.0001) samples. Tukey’s post hoc tests for pure water, PRP and WB samples showed significant differences between straight and single-loop, straight and double-loop and single-loop and double-loop strips. The *p*-values for all these comparisons were generally <0.0001. However, for WB samples, *p* = 0.0004 was found only between single-loop and double-loop strips.

In our previous study [25], we observed the highest fluid velocities very early in the capillary flow, with estimates from images captured at very short intervals within the first 3 s. In the light of the available data, more noise would be expected at these instantaneous velocities, because the very short time intervals and small changes in image capture timing or factors like strip bending can introduce additional noise compared to the estimated velocities, as shown in Figure 2c. This effect is more noticeable compared to the longer time intervals used for velocity estimation when the velocities are significantly lower. Consequently, we believe that the high level of noise observed is likely due to our lower confidence in the time differences between images during the early stages. This hypothesis can be tested using alternative image capture methods, such as high-resolution, high-frame-rate video cameras.

### 3.3. Loop Design Can Detect Lower Stimulus Concentrations by Increasing Stimulation Time without Affecting Capillary Force

**Figure 3:**
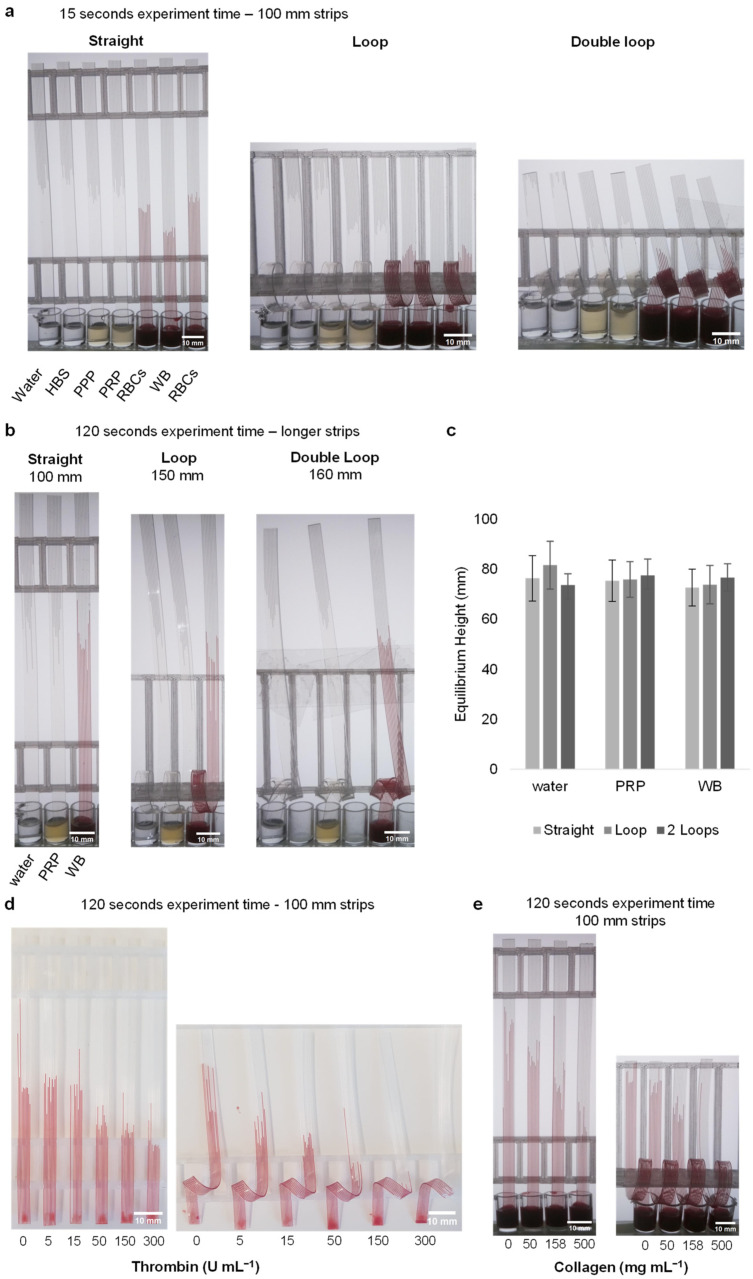
The capillary rise is unaffected by the loop, and impact of stimulation with thrombin and collagen can be measured. (**a**) Demonstration of the capillary rise of water, buffer and blood components in straight, loop- and double-loop-added dipsticks after 15 s. (**b**) An image of the strips before reaching equilibrium height. Strips of different sizes were used. (**c**) After 10 min, the equilibrium heights reached by water, plasma, and blood in the dipsticks with straight, loop, and double-loop configurations showed nearly the same values. Data indicates 10 replicate capillaries where error bars indicate ± SD from a single donor. (**d**) At the end of the 2 min experiment, stimulation was observed in straight and loop-added dipsticks loaded with thrombin (loaded with 0, 5, 15, 50, 150 and 300 U mL^−1^). Since blood at concentrations of 50 U mL^−1^ and above could not pass through the loop within the 2 min time frame, these concentrations are not included in the calculations. The images provided are representative examples, with similar results observed across four different donors (N = 4). (**e**) Stimulation was observed in straight and loop-added dipsticks loaded with collagen (loaded with 0, 50, 158 and 500 mg mL^−1^). The images are representative examples of three replicate collagen stimulation experiments (N = 3).

Straight, looped and double-looped strips of 100 mm each were dipped in water, buffer and blood components for 2 min. As the liquid properties of each sample were different, the velocity of the rise was also different. After 15 s, in the double-looped strips, although water and HBS reached the top of the strips, it was observed that plasma samples reached only half the distance and whole blood barely passed the looped section (Figure 3a). A similar situation was observed in single-loop strips. Higher resolution versions of these images, provided as Supplementary Data, clearly show that the individual capillary height can be determined (Figures S2–S4). At 15 s, the height values on the straight strip are as follows: 53.10 mm for pure water, 55.99 mm for HBS, 49.22 mm for PPP, 51.09 mm for PRP, 44.93 mm for RBCs and 36.57 mm for WB. In contrast, the height values on the loop strip are as follows: pure water 41.51 mm, HBS 43.35 mm, PPP 33.43 mm, PRP 33.71 mm and RBCs 26.17 mm. The height of WB could not be measured because it could not pass the loop zone. The distance values were, respectively, 84.96 mm, 88.67 mm, 76.48 mm, 76.81 mm and 68.97 mm. Distance could not be determined for WB for the same reason. The height values on the double-loop strip were 37.72 mm for pure water, 39.59 mm for HBS, 23.30 mm for PPP, 21.82 mm for PRP and 18.37 mm for RBCs. The distance values were 95.72 mm, 97.49 mm, 80.70 mm, 79.26 mm and 73.37 mm, respectively. The distance and height of WB could not be determined because it could not pass the loop region.

The surface tension and internal structure of capillary strips affect the velocity and height of liquid rise. To check that the loop does not affect the capillary force and thus equilibrium height, we measured height of liquids with different viscosities, densities, and surface tensions in straight and looped test strips for ultrapure water, PRP, and WB after 10 min (Figure 3b). The equilibrium height plots show that, as expected, the fluids approached equivalent endpoint heights within both straight and looped strips. After 10 min, the following heights were determined for straight, looped, and double looped strips: 76.3 ± 9.1, 81.6 ± 9.6 and 73.7 ± 4.5, respectively, for ultrapure water. The corresponding values for PRP were 75.4 ± 8.3, 75.9 ± 7.1 and 77.5 ± 6.6, while for WB, the values were 72.7 ± 7.4, 73.8 ± 7.7 and 76.7 ± 5.5 (Figure 3c). This indicates that capillary rise is not affected by the presence of loops, demonstrating that the capillary phenomenon remains consistent regardless of the loop configuration.

Stimulating WB with thrombin and collagen in microcapillaries caused a rapid and concentration-dependent decrease in height, compared to unstimulated blood. We observed similar responses across several donors. As blood flows through capillaries and comes into contact with the thrombin and collagen, we would anticipate an increase in viscosity over time after the triggering of coagulation and platelet activation. This stimulation will be expected to be uneven, with the highest viscosity gradient for coagulation at the top, where the blood is exposed to the stimuli for the longest duration. Increased thrombin concentrations lead to lower heights for both straight and looped capillaries. However, looped capillaries showed a greater difference compared to straight strip tests (Figure 3d). This was expected because the addition of loops was intended to slow the velocity and prolong stimulation times for coagulation and/or platelet activation to take place and increase viscosity. While the 5 and 15 U mL^−1^ thrombin-activated blood samples gave almost the same heights as unstimulated blood in straight strip tests, a difference was observed when activating blood in looped strips. Even the lowest concentration level of 5 U mL^−1^ showed a reduced rise in looped strips, while a significant effect was only observed in straight strips after 50 U mL^−1^. Although there were differences between 50, 150, and 300 U mL^−1^ concentrations, these differences did not exceed 4 mm. However, in looped strips, this difference reached up to 11 mm. In addition, when the length of the blood L(t) in the capillary was examined in looped strips, it was observed that there was more migration in the capillary compared to straight strips. The 150 and 300 U mL^−1^ thrombin concentrations in looped strips were not evaluated because the blood sample did not pass the loop area. Similar results were observed in collagen-loaded straight and looped strips (Figure 3e). Data could not be obtained for 500 mg mL^−1^ because the blood could not pass the loop in the 500 mg mL^−1^ collagen-loaded strip. The dip-stick tests work by allowing the blood sample to flow up vertically into a ~200-micron channel where the blood mixes with stimuli. When blood pathways are activated (including coagulation and thrombus formation, for example, by fibrin formation or platelet activation) this makes the blood become thicker with increased resistance to flow. The most important functional change in blood when activated is to stop flowing, which is how we prevent bleeding tissue or blood vessel injury. As the flow properties of blood change, we can measure them by monitoring the rate of flow in capillary devices.

Surprisingly, adding a simple loop to the vertical dipstick causes a significant improvement in how these assays perform. A longer path to flow provides a longer period of time for the blood flow properties to be measured whilst they change following the addition of stimulus. After dipping, the blood flows vertically upwards, but then around a loop. With the addition of the loop, the blood sample liquid flows far further before hydrostatic pressure increases. This allows for a faster flow for a longer period of time. This means we have the potential to measure changes in flow occurring much later by increasing the time scale of the stimulation process, and with lower doses of stimuli. Notably, the vertical flow in straight channels takes only a few seconds before slowing down. With the addition of a loop, this rapid flow continues for >30 s. Using this simple approach, we can stimulate blood for much longer whilst still measuring flow. Conveniently, the length of time that blood stimulation leads to changes in flow properties is also in the 3–30 s range, so straight vertical strips simply do not stimulate for long enough, yet looped channels do stimulate for a length of time that matches the time taken for the blood activation process that we need to measure.

Different channel geometries such as T channels, Y channels, trifurcation, serpentine channels and bend channels have been applied to microfluidic systems for WB plasma separation [14]. However, these studies have generally focused on blood plasma separation. Many researchers have used bend, curved and spiral microfluidic devices to separate plasma [15,18,22], RBCs [16,17,20,21] and platelets [19] from WB [14]. By advancing these studies and taking advantage of such geometries, platelet stimulation can be achieved. Our method shows that by controlling the flow conditions inside the microfluidic device, the stimulation time of blood and other parameters of the assay can be precisely controlled. It is also possible that loops can offer improved mixing compared to straight capillaries, with curvature leading to reduced diffusion distance and the increased transportation of the stimulus (thrombin/collagen) dried within the capillaries [6]. In our study, we found that the addition of loops allows for the better detection of blood stimulation responses even at lower thrombin concentrations compared to vertical microcapillaries. This approach has the potential to improve the accuracy and reliability of blood stimulation tests, reducing the additional costs associated with the use of high-concentration reagents. The addition of loops offers the ability to improve the measurement of blood activation in low-cost microfluidic devices by reducing the flow rate and extending the residence time of fluid in capillaries, without switching to more complex fabrication methods. This technology could contribute to a more precise assessment of the blood’s response to stimulation for both research and clinical applications. In particular, while blood clotting, fibrin formation and platelet activation by thrombin stimulation are measured by routine coagulation tests, the ability of the loops to adapt to the high viscosity changes in this process also offers significant advantages. For the validation of this technology, more stimuli are needed, and larger-scale studies need to be conducted using a wide variety of donors, both in healthy populations and in specific conditions known to change blood function. 

Overall, looped microcapillary testing has the potential to improve the measurement of blood activation. Triggering blood clotting by activating both fibrin formation and platelets with thrombin is commonly measured by routine clotting tests or by investigating the suitability of an anticoagulant drug for treatment, likely similar to those applied before surgical procedures. If increased sensitivity can be achieved by loop modification, which allows for longer stimulation times, it may provide several benefits such as minimizing time loss and reducing the potential for inaccuracies before emergency surgical procedures. Crucially, the addition of a loop allows the device flow characteristics to be modified without switching to a more complex, higher cost fabrication method, retaining the low-cost and simplicity of melt-extruded microcapillary devices.

## 4. Conclusions

Comparing the equilibrium height for straight and looped MCFs using water, plasma, and blood showed that the use of looped strips did not affect the capillary force. Using measurements of instantaneous capillary rise and equilibrium capillary height in straight and loop-added vertical hydrophilic microcapillaries, quantitative information was obtained on the changes in capillary flow of water, plasma and blood samples caused by loop addition. The observed mean superficial flow rates of capillary rise were evaluated as the best fit to pressure balance models. In this way, the effect of loop addition on flow dynamics for different liquids was analyzed in detail. Experiments have shown that loops create additional frictional resistances, which reduce the flow velocity and lead to longer residence times. This study demonstrates that data from microfluidic systems can be extended, and haemostasis dynamics can be measured on a larger scale using low-cost and open-source devices. Understanding the effects of loop addition on fluid dynamics provides important information for the optimization and development of microfluidic devices.

We found that the performance of low-cost extruded microfluidic dip-sticks used for blood function measurements can be enhanced by a simple addition: a “loop”. The loop introduces some valuable features of more complex and hard-to-manufacture “lab-on-a-chip” devices. The loop adds a “stimulation zone” where blood can respond to stimulus, prior to a vertical measurement stage. This ensures that coagulation and platelet activation can occur before flow ends. The effect of loops changes as fluid viscosity increases, with higher viscosity of whole blood also slowing the overall flow rate. Loops are useful for microsystems where large viscosity changes are likely, for example, in thrombin stimulation. This new method has the potential to improve the accuracy and sensitivity of blood stimulation assays in a large-scale population analysis.

## Data Availability

Data are contained within the article. The open source designs, software and details for the RMS imaging rig [25] are available from https://gitlab.com/ruyameltem/imaging_rig.

## Supplementary Materials

The following supporting information can be downloaded at: https://www.mdpi.com/article/10.3390/s24185870/s1, Figure S1: Schematic of the experimental setup. Figure S2: Figure 3a images with enlargement to show detail of individual capillaries. Figure S3: Figure 3b, enlarged view. Figure S4: Figure 3d,e images enlarged views.
