# Supplementary material for: Looping Flexible Fluoropolymer Microcapillary Film Extends Analysis Times for Vertical Microfluidic Blood Testing
Source: Sensors (Basel). 2024 Sep 10;24(18):5870. doi: 10.3390/s24185870 (PMC11436048; doi:10.3390/s24185870)
Supplement: Supplementary file 1 [file sensors-24-05870-s001.zip › sensors-3170903-supplementary.pdf]

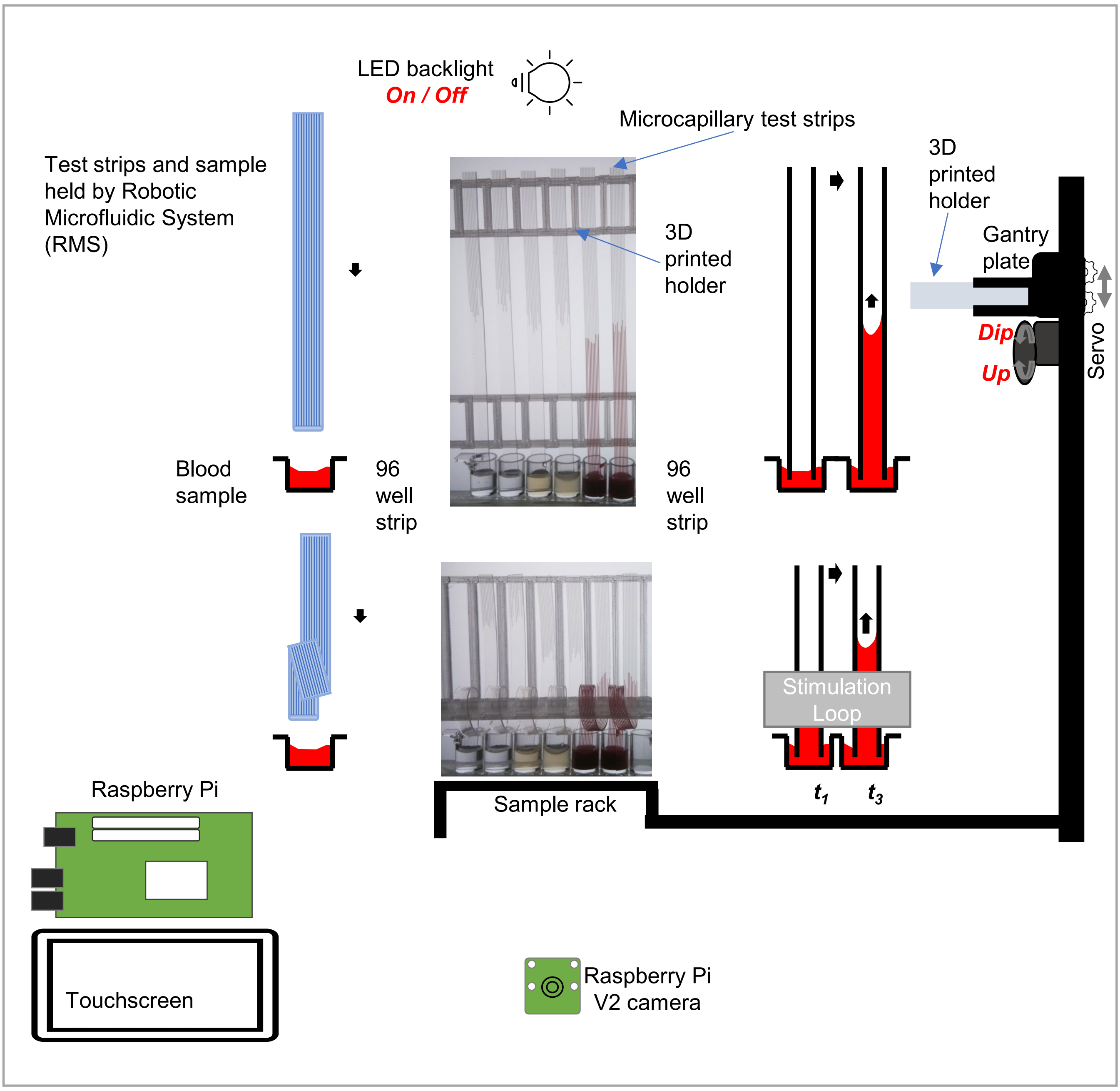

Figure S1: Schematic of the experimental setup.

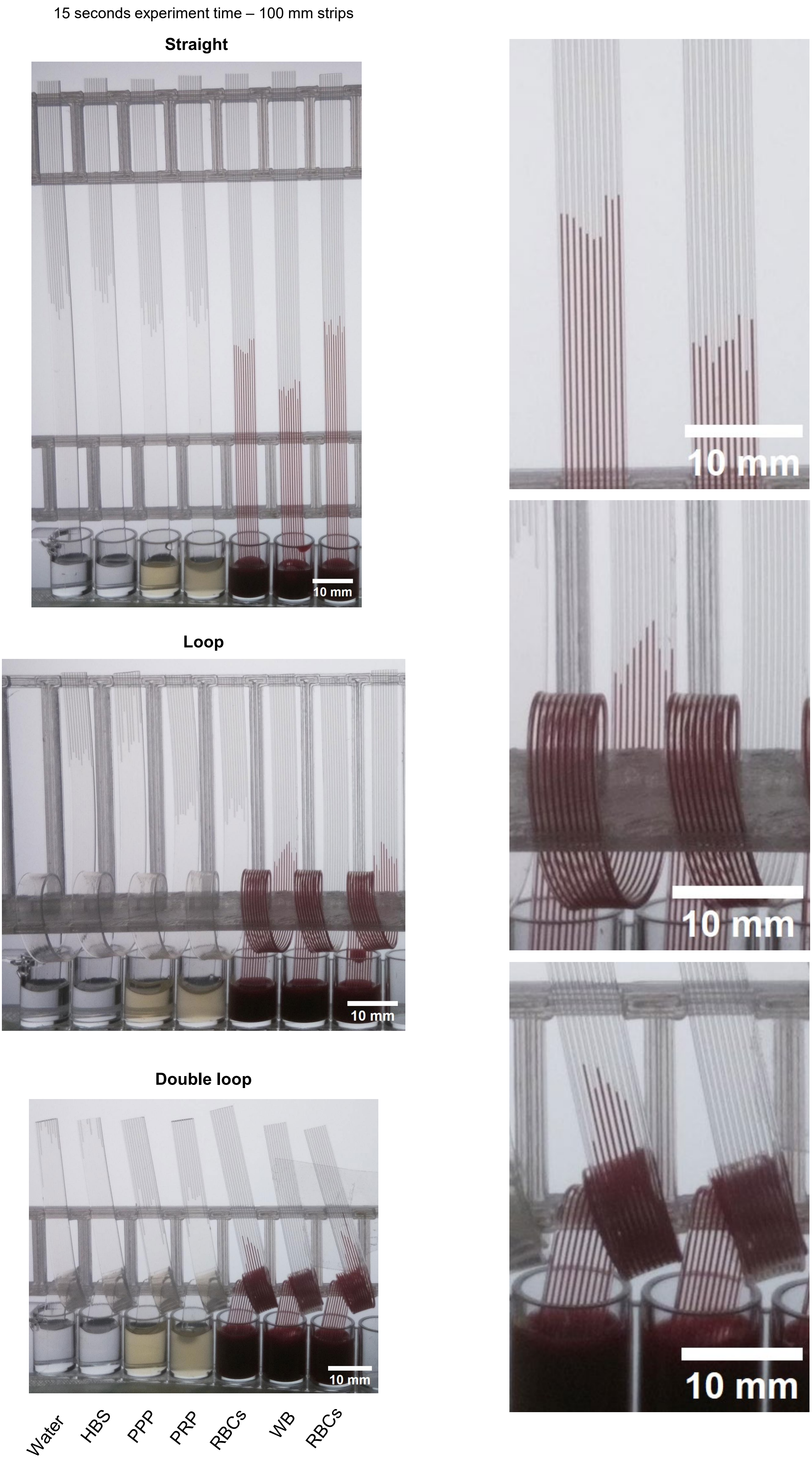

Figure S2: Figure 3a images with enlargement to show detail of individual capillaries.

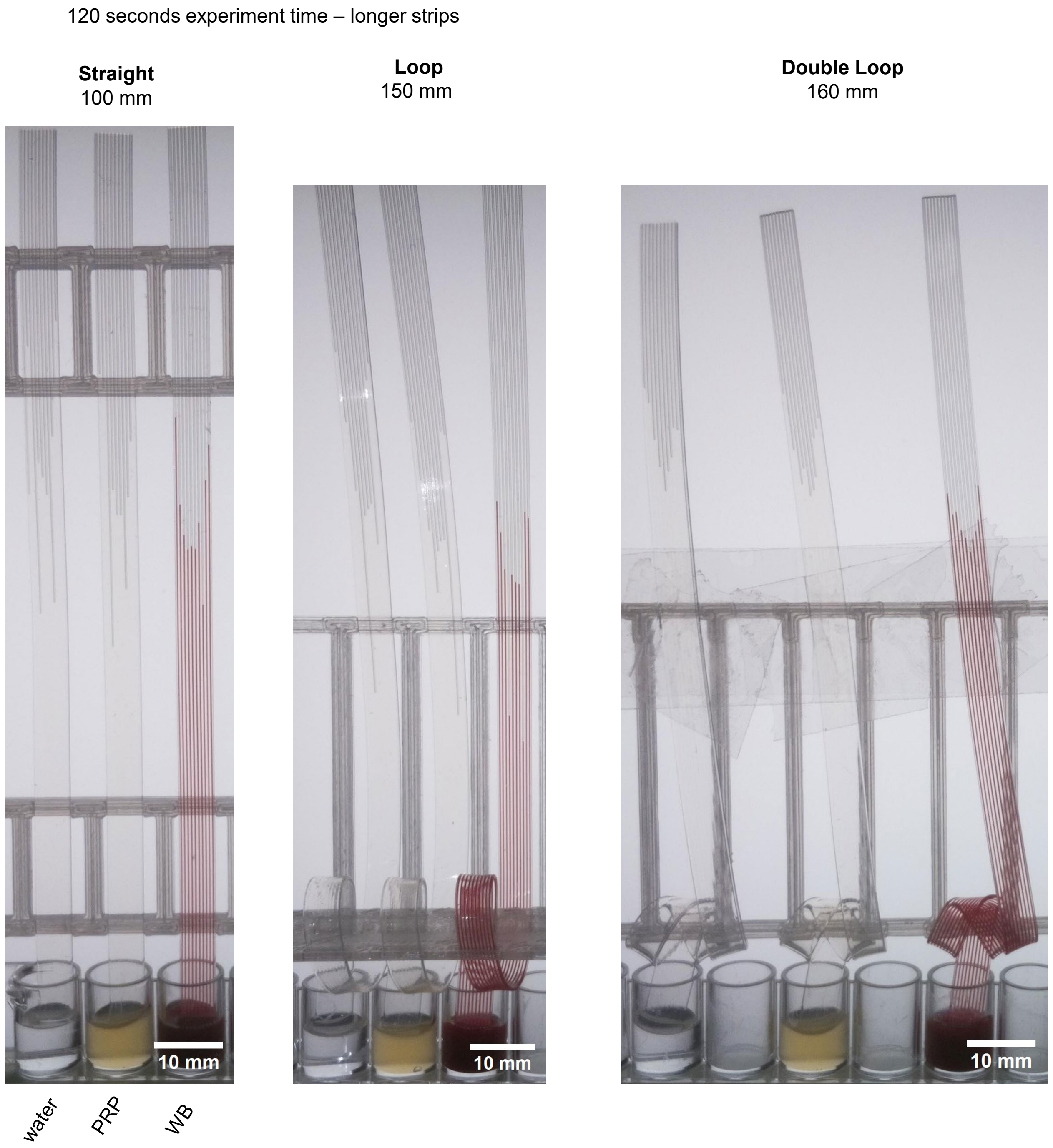

Figure S3: Figure 3b, enlarged view.

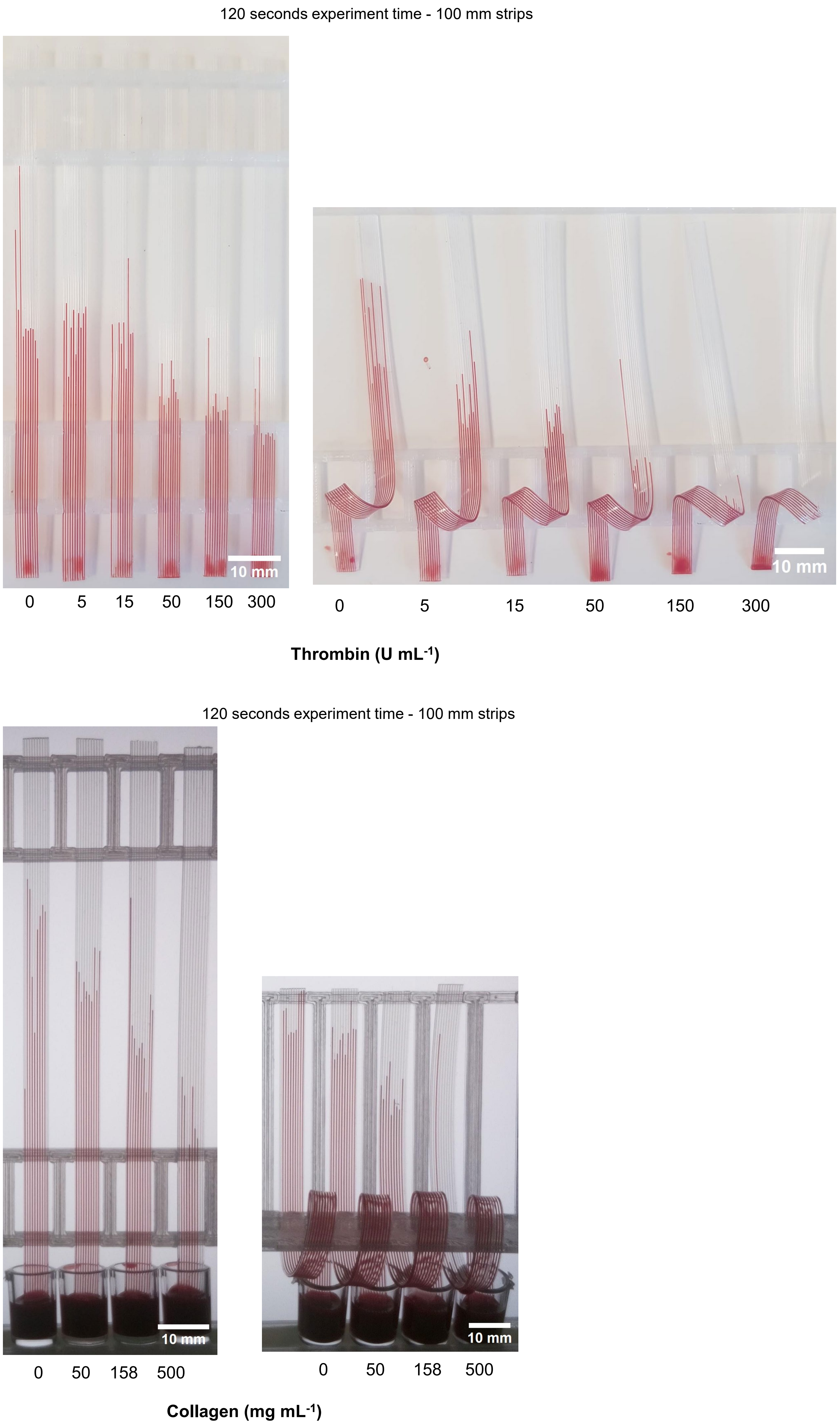

Figure S4: Figure 3d and 3e images enlarged views.
